# Toward a More Comprehensive Index of Youth Cigarette Smoking: Average Number of Cigarettes Smoked per Day among Students in the United States over Two Decades

**DOI:** 10.3390/ijerph18020478

**Published:** 2021-01-08

**Authors:** Michelle T. Bover Manderski, Cristine D. Delnevo, Kenneth E. Warner

**Affiliations:** 1 Rutgers Center for Tobacco Studies, New Brunswick, NJ 08901, USA; delnevo@rutgers.edu; 2 Department of Biostatistics & Epidemiology, Rutgers School of Public Health, Piscataway, NJ 08854, USA; 3 Department of Health Behavior, Society, and Policy, Rutgers School of Public Health, Piscataway, NJ 08854, USA; 4 Department of Health Management and Policy, University of Michigan School of Public Health, Ann Arbor, MI 48109, USA; kwarner@umich.edu

**Keywords:** cigarette smoking, youth, high school students, measurement, survey

## Abstract

Reliance on 30-day prevalence as the principal means of assessing trends in youth cigarette smoking may understate the magnitude of the decrease in youth smoking, because prevalence does not account for smoking frequency or intensity. We analyzed Youth Risk Behavior Survey (YRBS) data from 1997 through 2017 and estimated cigarette smoking prevalence (any smoking in the previous 30 days), frequency (number of smoking days in the previous 30 days), and intensity (cigarettes per day on smoking days). We calculated average cigarettes smoked per day (ACSD) as the product of frequency and intensity, divided by 30. We estimated ACSD among all high school students and by smoking frequency group (i.e., 1–5, 6–9, 10–19, 20–29, or 30 of the previous 30 days), sex, grade level, and race/ethnicity. Among US high school students, ACSD declined by 86.7% from 1997 to 2017, while prevalence declined by 75.8%. Within smoking frequency groups, smoking intensity remained similar over the two decades. However, changes in ACSD over time varied by race/ethnicity; ACSD increased among Hispanic and non-Hispanic Black daily smokers while it decreased among daily smokers of other race/ethnicity groups. ACSD declined more substantially than smoking prevalence over two decades but remained virtually unchanged within smoking frequency groups, indicating that changes in frequency, rather than intensity, drove this decline. Prevalence estimates alone understate the degree to which youth in the United States have rejected smoking, and racial/ethnic disparities in smoking intensity are hidden when we limit our lens to prevalence-only measures.

## 1. Introduction

Reductions in cigarette smoking constitute one of the greatest public health achievements in the United States in the past half-century [1]. Adult smoking prevalence has declined by two-thirds, from 43% in 1965 to 14% in 2018. Tobacco control—the diverse collection of public and private sector interventions intended to reduce smoking—is credited with having avoided 8 million premature deaths between 1964 and 2012 [1,2]. Less often recognized is the dramatic decline in smoking among youth in the United States (US). Since it peaked in the mid-1990s, middle and high school students’ 30-day smoking prevalence has fallen 85%. In 1996, more than a fifth of 8th graders had smoked in the past 30 days. In 2019, only 2.3% had done so. Two decades ago, more than a third of high school seniors had smoked in the past 30 days. In 2019 that figure had plummeted to 5.7% [3].

As impressive as these numbers may be, they may not fully capture the extent of the decline in cigarette smoking among America’s high school students. Prevalence estimates tell us how many young people smoke cigarettes, but they do not reflect the frequency or intensity of their smoking—how many days per month they smoke and how many cigarettes they consume during those days. For example, the 30-day prevalence measure fails to distinguish between one-time experimentation with cigarettes and cigarette smoking every day [4]. Research demonstrates that both the frequency and the intensity of students’ cigarette smoking have decreased over time [5,6], which cannot be captured by 30-day prevalence measures. However, while these declines are encouraging, future monitoring is warranted as some previous studies suggest that even light and intermittent smoking in high school can lead to heavier smoking in young adulthood [7,8].

Reliance on 30-day prevalence as the principal means of assessing trends in youth cigarette smoking may thusly understate the magnitude of the decrease in youth smoking. Recently one of us (K.E.W.) combined prevalence with measures of frequency and intensity to produce a new index of youth smoking, average cigarettes smoked per day (ACSD), using data from Monitoring the Future [9]. From the peak of youth smoking prevalence in the mid-1990s to 2016, the decrease in ACSD exceeded the decline in prevalence. In the present study, we assess this relationship using a different data set, the National Youth Risk Behavior Survey (YRBS), and additionally assess whether average cigarette smoking intensity has changed within smoking frequency groups (e.g., whether daily smokers still smoke the same amount per day).

## 2. Materials and Methods

### 2.1. Data Source

We analyzed 1997 (i.e., the peak of youth smoking in the US) through 2017 Youth Risk Behavior Survey (YRBS) data from the YRBS combined dataset, which is publicly available online [10,11]. YRBS is an ongoing nationally representative survey of high school students in the United States that is conducted by the Centers for Disease Control and Prevention (CDC) between February and May during odd-numbered years. This well-developed surveillance system has undergone numerous methods studies to ensure that the questionnaire produces valid and reliable estimates [12,13]. Additional details on survey design and sampling, data collection, and weighting are provided elsewhere [10]. This study was determined to not meet the definition of human subjects research and as such did not require Institutional Review Board approval.

### 2.2. Participants

Sample sizes and response rates vary by year (Supplementary Table S1). For example, in 1997 there were 16,272 participants with an overall response rate of 68.9%, and in 2017 there were 14,765 participants with an overall response rate of 60% [14,15]. Participant demographics and response rates for each YRBS administration are detailed elsewhere [16].

### 2.3. Measures

Two outcomes of interest in our study were the prevalence and frequency of cigarette smoking among youth. We defined “current smokers” as students who had smoked a cigarette on 1 or more of the 30 days preceding the survey. Current smokers were further categorized into five frequency subgroups: “rare smokers” (1–5 of the past 30 days), “infrequent smokers” (6–9 of the past 30 days), “moderate smokers” (10–19 of the past 30 days), “frequent smokers” (20–29 of the past 30 days), and “daily smokers” (all 30 of the past 30 days).

We estimated average cigarettes smoked per day (ACSD) in the past 30 days. Both the number of smoking days and the number of cigarettes per day were assessed by questions with categorical response options; to convert these to continuous variables for analysis, the midpoint of the selected category range was assigned. For example, a response of “3 to 5 days” was recoded as 4 days and a response of “2 to 5 cigarettes” was recoded as 3.5 cigarettes. We then calculated ACSD in the past 30 days as the product of the number of smoking days and the number of cigarettes smoked on smoking days, divided by 30.

Sex (response options: female or male) and grade level (response options: 9th grade, 10th grade, 11th grade, or 12th grade) questions were consistent across all years included in this analysis. However, the assessment of race and ethnicity in the YRBS has changed over time: in 1997, both race and ethnicity were assessed by a single question that allowed one response; from 1999 through 2005, race and ethnicity were assessed by a similar single question that allowed multiple responses; beginning in 2017, Hispanic ethnicity and race were assessed separately, with multiple responses allowed for race [15]. We utilized the 7-level “bridging” race/ethnicity variable provided in the 1991–2017 YRBS dataset [10] and collapsed “American Indian/Alaskan Native”, “Native Hawaiian/Pacific Islander”, and “Multiple Races (Non-Hispanic)” into one group, leaving five race/ethnicity groups for analysis: non-Hispanic White, non-Hispanic Black, Hispanic, non-Hispanic Asian, and non-Hispanic other.

### 2.4. Statistical Analyses

We estimated prevalence of current smoking and prevalence of each smoking frequency level, as well as mean ACSD overall, among current smokers, and within each smoking frequency subgroup, among all high school students and by sex, grade level, and race/ethnicity. We performed all analyses using SAS software version 9.4 (SAS Institute Inc., Cary, NC, USA) survey procedures, accounting for the complex sampling design of YRBS. Estimates were weighted to account for nonresponse and to be representative of US high school students (grades 9 through 12), and 95% confidence intervals were calculated via Taylor series linearization, using the weight and sampling variables provided with the YRBS dataset [10,17].

## 3. Results

**Figure 1:**
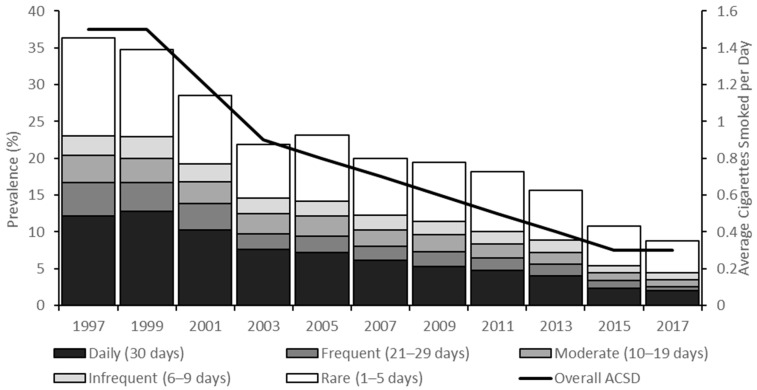
Prevalence and frequency of smoking among US high school students, National Youth Risk Behavior Survey 1997–2017.

**Figure 2:**
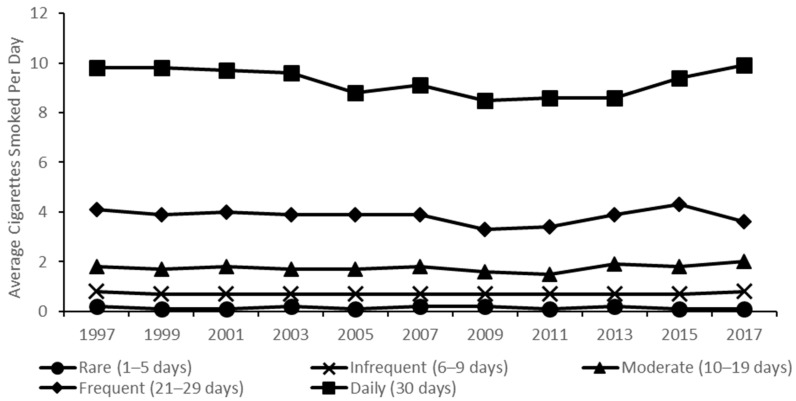
Average cigarettes smoked per day in the past 30 days by smoking frequency among US high school students, National Youth Risk Behavior Survey 1997–2017.

From 1997 to 2017, the prevalence of current smoking among high school students fell by 75.8% from 36.4% (95% confidence interval [CI]: 34.1, 38.6) to 8.8% (95% CI: 7.3, 10.6) (Figure 1 and Supplementary Table S2). Prevalence of rare, infrequent, moderate, frequent, and daily smoking also declined substantially during this time period, with the greatest decreases observed for frequent smoking (4.5% [95% CI: 3.8, 5.4] to 0.6% [95% CI: 0.4, 0.9], an 86.7% decrease) and daily smoking (12.2% [95% CI: 10.6, 14.0] to 2.0% [95% CI: 1.4, 2.9], an 83.6% decrease). Meanwhile, a greater decline occurred for the estimated average number of cigarettes smoked per day (ACSD) for all students, by 86.7% (1.5 cigarettes in 1997 [95% CI: 1.3, 1.7] to 0.2 cigarettes in 2017 [95% CI: 0.2, 0.3]). Among current smokers, ACSD declined by 29.3% (4.1 cigarettes [95% CI: 3.7, 4.5] to 2.9 cigarettes [95% CI: 2.5, 3.4]) (Figure 1 and Supplementary Table S3). However, there were no significant changes in smoking intensity over this period within frequency subgroups (Figure 2 and Supplementary Table S3). For example, daily smokers consumed about a half pack (10 cigarettes) per day in both 1997 and 2017, with minimal variation over the two decades.

The percent decline in current smoking prevalence from 1997 to 2017 was slightly higher for females than males (77.5% vs. 74.0%, respectively) and decreased with grade level (Table 1). By race/ethnicity, non-Hispanic Asians experienced the greatest percent declines (89.6%), while non-Hispanic White students experienced the smallest percent decrease (72.0%). Percent changes in daily smoking prevalence similarly declined with grade level (90.4% for 9th graders, 76.2% for 12th graders) but were slightly greater for males than females and were lowest for non-Hispanic Black students (80.0%).

Despite marked declines in both current and daily smoking, non-Hispanic Black smokers were the only demographic group to experience an increase in ACSD from 1997 to 2017 (Table 2). Although overall smoking prevalence for this group was lower (Table 1), among current smokers in 2017 non-Hispanic Black youth smoked more cigarettes per day than their non-Hispanic White and Hispanic counterparts (Table 2). Additionally, although ACSD among daily smokers of all races and ethnicities was virtually unchanged over this time period, ACSD increased by 52.6% (from 7.8 [95% CI: 6.5, 9.2] to 11.9 [95% CI: 9.3, 14.5]) among Hispanic daily smokers and by 31.1% among non-Hispanic Black daily smokers, while other race/ethnicity groups experienced modest declines.

## 4. Discussion

Prevalence of current smoking among high school students decreased substantially (by 75.8%) from 1997 to 2017, with frequent and daily smoking estimates exhibiting the greatest declines. Overall, ACSD declined even more (by 86.7%) over the same two decades but remained virtually unchanged within each smoking frequency group, indicating that changes in frequency (number of smoking days per month), rather than intensity (cigarettes per day), drove the decline in overall ACSD. However, despite comparable declines in smoking prevalence over this time period, ACSD increased among non-Hispanic Black and Hispanic daily smokers, revealing a disparity that is not evident when only assessing prevalence.

Importantly, our findings are quite consistent with the previous analysis of Monitoring the Future data by Warner, which found significant declines of 71.3% and 83.9% in smoking prevalence and ACSD, respectively, among US high school seniors [9]. In addition to demonstrating consistency across datasets, we further expand upon this previous work by demonstrating that the observed trends hold across different datasets and by extending the grade levels of inclusion from two (10th and 12th grade) to four (9th through 12th grade) grades. We additionally estimated ACSD within smoking frequency groups; in contrast to overall estimates, ACSD within these subgroups remained constant over the study period, indicating that smoking intensity is not driving the overall decline.

An analysis of YRBS data from 1991 through 2009 by Jones and colleagues observed declines in heavy smoking and increases in infrequent smoking among current smokers [5]. Our observed decline in ACSD overall is consistent with their results, but our finding that ACSD did not decline within smoking frequency groups suggests that cigarettes per day alone, without accounting for number of smoking days per month, is not sufficient for fully capturing youth smoking trends.

Although all racial/ethnic groups experienced substantial declines in current and daily smoking prevalence, ACSD increased among Black youth who smoke and among Black and Hispanic students who smoke every day. This may suggest that behavioral and policy interventions aimed at reducing youth exposure to tobacco are not equally effective across racial/ethnic groups. However, given that prevalence was already (and remains) lower among Black students, it is also possible that we are observing a regression to the mean over time. Additional studies to assess and address the disparate changes in ACSD over time are therefore needed.

The present study is subject to several limitations common to survey research. First, data are self-reported and subject to misreporting, and the direction and magnitude of misreporting may have changed over time. For example, youth who smoked cigarettes in 2017 may have been more reluctant to report their behavior than youth in 1997, due to changes in social norms. Second, YRBS is a school-based survey; thus, absent, suspended, and expelled students are not included, potentially biasing estimates downward. Third, changes in assessment of race and ethnicity over time limited our ability to analyze more refined subgroups. Finally, we did not account for use of other tobacco products or polytobacco use, both of which remain prevalent as cigarette smoking declines [18].

Despite these limitations, this study was strengthened by including 20 years of nationally representative data, permitting assessment of long-term population trends. We demonstrated that the current standards in youth tobacco surveillance are inadequate for completely capturing tobacco use patterns in this population. Indeed, prevalence estimates alone understate the degree to which US youth have rejected smoking, and racial/ethnic disparities in smoking intensity are hidden when we limit our lens to prevalence-only measures. Given that increased smoking frequency during adolescence may predict adult smoking [19], declines in frequent and daily high school smoking may have a greater impact than an equal decrease in infrequent smoking. However, research also suggests that even light and infrequent smoking in high school is associated with heavier smoking in young adulthood [7,8]; as such, continued monitoring of youth smoking frequency and intensity is needed to better understand young adult and adult smoking behaviors.

As the tobacco landscape continues to change, with new regulations and the emergence of new products, adolescent exposure to tobacco has changed as well. About two in five high school students who used tobacco or alternative nicotine products in 2018 had used two or more products, and cigarettes are no longer the most prevalent product used by US youth [18]. Indeed, the marked declines in ACSD that we observed in this study could conceivably reflect supplementation with other nicotine products that are not directly comparable to cigarettes in terms of risk (e.g., smoking cigarettes less frequently but using alternative products instead). Future studies to develop surveillance measures that combine frequency and intensity of noncigarette product use, as well as use of multiple products, are warranted in order to more fully understand youth tobacco product use.

## 5. Conclusions

These results demonstrate that the current standards in youth tobacco surveillance are inadequate for completely capturing tobacco use patterns in this population. Average cigarettes smoked per day (ACSD) combines smoking frequency and intensity to provide a more comprehensive measure of youth tobacco use.

## Figures and Tables

**Table 1:** Prevalence of current and daily smoking among US high school students by sex, grade, and race/ethnicity, National Youth Risk Behavior Survey 1997–2017.

	1997		2001		2005		2009		2013		2017		1997 to 2017
	%	(95% CI)	%	(95% CI)	%	(95% CI)	%	(95% CI)	%	(95% CI)	%	(95% CI)	% Change
Current Smoking	36.3	(34.1, 38.6)	28.5	(26.5, 30.6)	23.0	(20.7, 25.4)	19.4	(17.9, 21.0)	15.7	(13.6, 18.0)	8.8	(7.2, 10.6)	−75.8%
Sex													
Female	34.7	(31.9, 37.6)	27.7	(25.7, 29.9)	23.0	(20.4, 25.7)	19.1	(17.3, 21.0)	15.0	(12.5, 17.8)	7.8	(6.1, 9.9)	−77.5%
Male	37.7	(35.0, 40.5)	29.2	(26.7, 31.9)	22.9	(20.8, 25.3)	19.8	(17.8, 21.9)	16.4	(14.3, 18.6)	9.8	(8.3, 11.5)	−74.0%
Grade													
9th Grade	33.4	(28.5, 38.8)	23.9	(21.2, 26.9)	19.7	(17.5, 22.1)	13.5	(12.0, 15.3)	10.2	(8.5, 12.1)	5.2	(3.9, 7.0)	−84.4%
10th Grade	35.3	(31.3, 39.6)	26.9	(23.9, 30.2)	21.4	(18.5, 24.7)	18.3	(15.9, 20.9)	13.2	(11.3, 15.3)	7.6	(6.2, 9.2)	−78.5%
11th Grade	36.6	(33.0, 40.3)	29.8	(26.2, 33.6)	24.3	(21.3, 27.6)	22.3	(19.7, 25.1)	21.1	(16.8, 26.1)	9.5	(7.5, 11.9)	−74.0%
12th Grade	39.6	(34.8, 44.5)	35.2	(31.2, 39.4)	27.6	(24.1, 31.4)	25.2	(22.6, 28.1)	19.2	(16.5, 22.2)	13.4	(11.0, 16.1)	−66.2%
Race/Ethnicity													
NH White	39.7	(37.4, 42.1)	31.9	(29.6, 34.3)	25.9	(23.0, 29.1)	22.5	(20.1, 25.1)	18.6	(15.8, 21.8)	11.1	(9.0, 13.5)	−72.0%
NH Black	22.7	(19.1, 26.7)	14.7	(12.1, 17.8)	12.9	(11.2, 14.8)	9.5	(8.2, 11.0)	8.3	(6.4, 10.6)	4.4	(3.2, 5.8)	−80.6%
Hispanic	34.0	(31.3, 36.8)	26.6	(22.5, 31.1)	22.0	(18.8, 25.7)	18.0	(16.1, 20.2)	14.0	(11.3, 17.3)	7.0	(5.8, 8.6)	−79.4%
NH Asian	21.1	(16.7, 26.2)	15.2	(10.9, 20.7)	9.9	(6.7, 14.4)	7.5	(5.3, 10.4)	10.3	(6.4, 16.3)	2.2	(1.2, 4.0)	−89.6%
NH Other	44.4	(35.3, 54.0)	31.9	(27.4, 36.8)	25.9	(21.0, 31.5)	22.7	(18.8, 27.1)	14.4	(10.7, 19.0)	7.9	(5.5, 11.1)	−82.2%
Daily Smoking	12.2	(10.6, 14.0)	10.3	(9.0, 11.6)	7.1	(6.0, 8.4)	5.3	(4.6, 6.1)	4.0	(3.1, 5.2)	2.0	(1.4, 2.8)	−83.6%
Sex													
Female	11.3	(9.6, 13.2)	9.6	(8.3, 11.1)	6.9	(5.7, 8.4)	4.6	(3.7, 5.6)	3.8	(2.6, 5.6)	2.0	(1.3, 3.1)	−82.3%
Male	12.9	(10.6, 15.7)	10.9	(9.5, 12.5)	7.3	(6.2, 8.6)	6.0	(5.2, 6.7)	4.2	(3.4, 5.4)	2.0	(1.5, 2.7)	−84.5%
Grade													
9th Grade	9.4	(6.6, 13.2)	6.2	(4.6, 8.3)	5.2	(3.9, 6.7)	3.3	(2.6, 4.2)	2.2	(1.5, 3.2)	0.9	(0.6, 1.6)	−90.4%
10th Grade	10.2	(8.7, 11.9)	9.2	(7.9, 10.7)	5.8	(4.4, 7.5)	4.0	(3.1, 5.1)	2.9	(2.0, 4.2)	1.4	(0.9, 2.3)	−86.3%
11th Grade	14.1	(11.5, 17.1)	12.2	(10.0, 14.7)	8.0	(6.4, 10.0)	6.1	(5.1, 7.2)	5.1	(3.6, 7.3)	2.2	(1.4, 3.6)	−84.4%
12th Grade	14.7	(12.0, 17.8)	15.1	(12.4, 18.3)	10.1	(8.2, 12.4)	8.4	(7.1, 10.0)	6.1	(4.7, 7.9)	3.5	(2.4, 5.0)	−76.2%
Race/Ethnicity													
NH White	14.4	(12.6, 16.5)	12.9	(11.4, 14.6)	8.6	(7.3, 10.2)	6.9	(5.9, 8.2)	5.6	(4.2, 7.4)	2.6	(1.8, 3.7)	−81.9%
NH Black	5.5	(4.1, 7.3)	3.5	(2.3, 5.3)	3.1	(1.9, 4.9)	1.5	(1.0, 2.2)	1.7	(1.1, 2.8)	1.1	(0.5, 2.2)	−80.0%
Hispanic	7.3	(5.2, 10.1)	4.7	(3.6, 6.1)	4.7	(3.4, 6.5)	3.0	(2.4, 3.8)	1.9	(1.4, 2.5)	1.3	(0.9, 1.9)	−82.2%
NH Asian	5.2	(3.1, 8.4)	4.2	(2.1, 8.3)	3.2	(1.4, 6.9)	2.2	(1.2, 4.2)	1.6	(0.5, 5.1)	0.3	(0.1, 1.1)	−94.2%
NH Other	16.0	(9.1, 26.6)	9.0	(6.3, 12.7)	9.4	(6.2, 14.0)	5.5	(3.4, 8.7)	3.6	(1.9, 6.7)	2.2	(1.1, 4.3)	−86.3%

Note: See Supplemental Table S1 for an expanded data table including all years and smoking frequency groups. Abbreviations: CI, confidence interval; NH, non-Hispanic.

**Table 2:** Average number of cigarettes smoked per day among current and daily smoking US high school students by sex, grade, and race/ethnicity, National Youth Risk Behavior Survey 1997–2017.

	1997		2001		2005		2009		2013		2017		1997 to 2017
	%	(95% CI)	%	(95% CI)	%	(95% CI)	%	(95% CI)	%	(95% CI)	%	(95% CI)	% Change
Current Smoking	4.1	(3.7, 4.5)	4.3	(3.9, 4.7)	3.5	(3.1, 3.8)	3.0	(2.8, 3.2)	2.9	(2.5, 3.4)	2.9	(2.5, 3.4)	−29.3%
Sex													
Female	3.6	(3.2, 4)	3.8	(3.3, 4.2)	3.0	(2.6, 3.4)	2.4	(2.1, 2.7)	2.5	(1.8, 3.2)	2.7	(2.1, 3.3)	−25.0%
Male	4.5	(3.9, 5)	4.8	(4.4, 5.3)	3.9	(3.5, 4.2)	3.6	(3.2, 3.9)	3.3	(2.9, 3.7)	3.0	(2.5, 3.4)	−33.3%
Grade													
9th Grade	3.6	(2.8, 4.4)	3.2	(2.5, 3.8)	2.9	(2.3, 3.4)	2.9	(2.3, 3.4)	2.7	(2.0, 3.5)	2.2	(1.6, 2.8)	−38.9%
10th Grade	3.5	(2.8, 4.1)	4.0	(3.5, 4.5)	2.8	(2.3, 3.2)	2.6	(2.2, 2.9)	2.7	(2.0, 3.4)	2.7	(1.7, 3.7)	−22.9%
11th Grade	4.4	(4.0, 4.9)	4.6	(4.0, 5.1)	3.8	(3.2, 4.3)	3.0	(2.7, 3.3)	2.6	(2.1, 3.2)	2.5	(1.7, 3.3)	−43.2%
12th Grade	4.6	(3.6, 5.6)	5.3	(4.6, 6.0)	4.2	(3.7, 4.6)	3.5	(3.1, 3.8)	3.5	(2.6, 4.3)	3.4	(2.6, 4.1)	−26.1%
Race/Ethnicity													
NH White	4.5	(4.0, 4.9)	4.8	(4.3, 5.3)	3.8	(3.4, 4.1)	3.2	(3.0, 3.5)	3.4	(2.9, 4.0)	2.8	(2.3, 3.3)	−37.8%
NH Black	2.3	(1.8, 2.8)	2.6	(1.9, 3.3)	2.0	(1.3, 2.6)	2.2	(1.3, 3.1)	1.9	(1.3, 2.6)	3.3	(1.7, 4.9)	43.5%
Hispanic	2.5	(1.9, 3.1)	2.4	(1.8, 2.9)	2.5	(1.8, 3.2)	2.1	(1.7, 2.4)	1.9	(1.5, 2.3)	2.4	(1.6, 3.3)	−4.0%
NH Asian	3.1	(2.2, 3.9)	3.5	(1.9, 5.1)	2.7	(1.0, 4.5)	5.2	(2.5, 7.9)	3.4	(0.0, 6.8)	1.7	(0.0, 3.3)	−45.2%
NH Other	5.1	(3.0, 7.1)	3.2	(2.3, 4.2)	4.0	(3.1, 5.0)	2.9	(1.9, 3.8)	1.7	(1.1, 2.3)	3.6	(1.7, 5.5)	−29.4%
Daily Smoking (all 30 days)	9.8	(9.2, 10.4)	9.7	(9.3, 10.1)	8.8	(8.3, 9.4)	8.5	(7.9, 9.1)	8.6	(7.7, 9.4)	9.9	(8.8, 10.9)	1.0%
Sex													
Female	8.8	(8.2, 9.4)	8.8	(8.3, 9.4)	7.7	(7.0, 8.5)	7.3	(6.7, 7.8)	7.2	(6.0, 8.4)	8.3	(7.4, 9.2)	−5.7%
Male	10.5	(9.5, 11.5)	10.5	(9.9, 11.1)	9.9	(9.3, 10.4)	9.5	(8.7, 10.4)	9.8	(8.9, 10.7)	11.1	(9.5, 12.8)	5.7%
Grade													
9th Grade	10.1	(8.7, 11.5)	9.0	(8.2, 9.9)	8.5	(7.4, 9.6)	9.2	(7.8, 10.6)	9.9	(8.4, 11.4)	7.8	(5.6, 9.9)	−22.8%
10th Grade	9.1	(8.1, 10.1)	9.6	(8.7, 10.5)	7.8	(6.8, 8.8)	8.4	(7.2, 9.6)	9.1	(7.2, 10.9)	11.0	(8.5, 13.5)	20.9%
11th Grade	9.4	(8.8, 10.1)	9.5	(8.8, 10.3)	9.0	(8.0, 10.0)	8.7	(7.8, 9.6)	7.6	(6.4, 8.9)	7.9	(6.1, 9.7)	−16.0%
12th Grade	10.3	(9.2, 11.4)	10.2	(9.6, 10.7)	9.4	(8.5, 10.3)	8.2	(7.4, 9.0)	8.5	(6.8, 10.3)	10.6	(9.3, 11.9)	2.9%
Race/Ethnicity													
NH White	9.9	(9.4, 10.5)	9.8	(9.4, 10.3)	9.1	(8.6, 9.6)	8.2	(7.6, 8.7)	8.7	(7.9, 9.6)	8.8	(7.4, 10.3)	−11.1%
NH Black	7.4	(6.0, 8.7)	8.7	(7.1, 10.4)	6.1	(5.2, 7.1)	10.0	(6.3, 13.6)	6.2	(4.4, 8.0)	9.7	(6.9, 12.6)	31.1%
Hispanic	7.8	(6.5, 9.2)	8.9	(7.5, 10.3)	8.9	(6.4, 11.3)	9.6	(7.8, 11.4)	9.7	(7.8, 11.5)	11.9	(9.3, 14.5)	52.6%
NH Asian	9.5	(6.1, 12.8)	9.8	(5.7, 13.8)	6.5	(2.1, 10.8)	14.1	(10.7, 17.5)	16.8	(12.3, 21.3)	8.0	(8.0, 8.0)	−15.8%
NH Other	12.4	(9.9, 14.8)	8.6	(7.1, 10.1)	9.0	(7.2, 10.8)	8.6	(6.5, 10.7)	5.1	(2.0, 8.2)	11.7	(8.9, 14.4)	−5.6%

Note: See Supplementary Table S3 for an expanded data table including all years and smoking frequency groups. Abbreviations: CI, confidence interval; NH, non-Hispanic.

## Data Availability

The data analyzed for this study are publicly available at https://www.cdc.gov/healthyyouth/data/yrbs/index.htm.

## Supplementary Materials

The following are available online at https://www.mdpi.com/1660-4601/18/2/478/s1. Table S1. School, Student, and Overall Response Rates by Year, Youth Risk Behavior Survey, 1997–2017. Table S2: Prevalence of current, rare, infrequent, moderate, frequent, and daily smoking among high school students by sex, grade, and race/ethnicity, National Youth Risk Behavior Survey 1997–2017. Table S3: Average cigarettes smoked per day among current, rare, infrequent, moderate, frequent, and daily smoking high school students by sex, grade, and race/ethnicity, National Youth Risk Behavior Survey 1997–2017.
