# Supplementary material for: Toward a More Comprehensive Index of Youth Cigarette Smoking: Average Number of Cigarettes Smoked per Day among Students in the United States over Two Decades
Source: Int J Environ Res Public Health. 2021 Jan 8;18(2):478. doi: 10.3390/ijerph18020478 (PMC7827857; doi:10.3390/ijerph18020478)
Supplement: Supplementary file 1 [file ijerph-18-00478-s001.pdf]

Table S1. School, Student, and Overall Response Rates by Year, Youth Risk Behavior Survey, 1997 – 2017

	1997	1999	2001	2003	2005	2007	2009	2011	2013	2015	2017
School Response Rate	79%	77%	75%	81%	78%	81%	81%	81%	77%	69%	75%
Student Response Rate	87%	86%	83%	83%	86%	84%	88%	87%	88%	86%	81%
Overall Response Rate	69%	66%	63%	67%	67%	68%	71%	71%	68%	60%	60%

Source: Centers for Disease Control and Prevention. National Youth Risk Behavior Survey Data User's Guides, 1997-2017. Available at https://www.cdc.gov/healthyyouth/data/yrbs/data.htm

Table S2. Prevalence of current, rare, infrequent, moderate, frequent, and daily smoking among high school students, by sex, grade, and race/ethnicity, National Youth Risk Behavior Survey 1997-2017

	1997		1999		2001		2003		2005		2007		2009		2011		2013		2015		2017	
	%	(95% CI)	%	(95% CI)	%	(95% CI)	%	(95% CI)	%	(95% CI)	%	(95% CI)	%	(95% CI)	%	(95% CI)	%	(95% CI)	%	(95% CI)	%	(95% CI)
Current Smoking	36.3	(34.1, 38.6)	34.8	(32.4, 37.3)	28.5	(26.5, 30.6)	21.9	(19.8, 24.1)	23	(20.7, 25.4)	20	(17.7, 22.5)	19.4	(17.9, 21)	18	(16.7, 19.4)	15.7	(13.6, 18)	10.8	(9.4, 12.3)	8.8	(7.2, 10.6)
Sex																						
Female	34.7	(31.9, 37.6)	34.9	(32.3, 37.6)	27.7	(25.7, 29.9)	21.9	(19.3, 24.8)	23	(20.4, 25.7)	18.7	(16.5, 21)	19.1	(17.3, 21)	16.1	(14.6, 17.8)	15	(12.5, 17.8)	9.7	(8.1, 11.7)	7.8	(6.1, 9.9)
Male	37.7	(35, 40.5)	34.7	(31.9, 37.6)	29.2	(26.7, 31.9)	21.8	(19.8, 24)	22.9	(20.8, 25.3)	21.3	(18.4, 24.5)	19.8	(17.8, 21.9)	19.9	(18.2, 21.6)	16.4	(14.3, 18.6)	11.8	(10.5, 13.3)	9.8	(8.3, 11.5)
Grade																						
9th Grade	33.4	(28.5, 38.8)	27.6	(24.1, 31.5)	23.9	(21.2, 26.9)	17.4	(15.1, 20)	19.7	(17.5, 22.1)	14.3	(12, 17)	13.5	(12, 15.3)	13	(11.2, 15.1)	10.2	(8.5, 12.1)	7.6	(5.9, 9.7)	5.2	(3.9, 7)
10th Grade	35.3	(31.3, 39.6)	34.7	(32.3, 37.1)	26.9	(23.9, 30.2)	21.8	(19, 24.8)	21.4	(18.5, 24.7)	19.6	(16.7, 22.7)	18.3	(15.9, 20.9)	15.6	(13.8, 17.6)	13.2	(11.3, 15.3)	8.8	(7, 11)	7.6	(6.2, 9.2)
11th Grade	36.6	(33, 40.3)	36	(33.1, 39.1)	29.8	(26.2, 33.6)	23.6	(20.6, 26.9)	24.3	(21.3, 27.6)	21.6	(18.5, 25.1)	22.3	(19.7, 25.1)	19.3	(17, 21.7)	21.1	(16.8, 26.1)	13.1	(11.4, 14.9)	9.5	(7.5, 11.9)
12th Grade	39.6	(34.8, 44.5)	42.8	(37.3, 48.4)	35.2	(31.2, 39.4)	26.2	(23.5, 29.2)	27.6	(24.1, 31.4)	26.5	(22.6, 30.7)	25.2	(22.6, 28.1)	25.1	(23.3, 27.1)	19.2	(16.5, 22.2)	14.1	(11.8, 16.7)	13.4	(11, 16.1)
Race/Ethnicity																						
NH White	39.7	(37.4, 42.1)	38.6	(35.5, 41.8)	31.9	(29.6, 34.3)	24.9	(22.5, 27.4)	25.9	(23, 29.1)	23.2	(20.5, 26.1)	22.5	(20.1, 25.1)	20.3	(18.5, 22.1)	18.6	(15.8, 21.8)	12.4	(10.1, 15.1)	11.1	(9, 13.5)
NH Black	22.7	(19.1, 26.7)	19.7	(15.9, 24.2)	14.7	(12.1, 17.8)	15.1	(12.5, 18.1)	12.9	(11.2, 14.8)	11.6	(9.6, 14.1)	9.5	(8.2, 11)	10.5	(8.5, 12.9)	8.3	(6.4, 10.6)	6.5	(4.9, 8.6)	4.4	(3.2, 5.8)
Hispanic	34	(31.3, 36.8)	32.7	(29.1, 36.5)	26.6	(22.5, 31.1)	18.4	(16.2, 20.8)	22	(18.8, 25.7)	16.7	(13.6, 20.2)	18	(16.1, 20.2)	17.5	(15.4, 19.8)	14	(11.3, 17.3)	9.2	(8, 10.7)	7	(5.8, 8.6)
NH Asian	21.1	(16.7, 26.2)	22.9	(17.3, 29.7)	15.2	(10.9, 20.7)	13	(9.5, 17.5)	9.9	(6.7, 14.4)	9.9	(6.1, 15.7)	7.5	(5.3, 10.4)	7.6	(5.3, 10.9)	10.3	(6.4, 16.3)	7	(4.3, 11.1)	2.2	(1.2, 4)
NH Other	44.4	(35.3, 54)	35.5	(31.2, 40.1)	31.9	(27.4, 36.8)	20.7	(15.3, 27.3)	25.9	(21, 31.5)	23.5	(16.2, 32.8)	22.7	(18.8, 27.1)	21	(17.2, 25.3)	14.4	(10.7, 19)	13.7	(10.7, 17.5)	7.9	(5.5, 11.1)
Rare Smoking (1-5 days)	13.4	(12.4, 14.6)	11.9	(10.9, 12.9)	9.3	(8.6, 10.1)	7.3	(6.6, 8.2)	8.9	(8.1, 9.7)	7.7	(6.9, 8.6)	8.1	(7.4, 9)	8.1	(7.4, 8.9)	6.7	(5.9, 7.6)	5.4	(4.8, 6.1)	4.3	(3.7, 4.9)
Sex																						
Female	12.6	(11.1, 14.2)	12.4	(10.8, 14)	9.7	(8.8, 10.7)	7.7	(6.8, 8.7)	9.1	(8.1, 10.2)	7.4	(6.6, 8.4)	8.8	(7.9, 9.7)	7.9	(7.1, 8.7)	6.5	(5.6, 7.5)	4.6	(3.8, 5.6)	3.7	(3.1, 4.5)
Male	14.1	(12.7, 15.7)	11.4	(10.2, 12.6)	8.9	(7.8, 10.2)	7	(6.1, 8)	8.7	(7.8, 9.6)	8	(6.8, 9.3)	7.5	(6.5, 8.7)	8.3	(7.3, 9.4)	7	(6, 8.1)	6.2	(5.4, 7)	4.8	(4.2, 5.5)
Grade																						
9th Grade	13.2	(11.3, 15.3)	11.6	(9.7, 13.7)	9.8	(8.7, 11.1)	6.7	(5.5, 8.2)	8.3	(7.2, 9.5)	6.7	(5.6, 8)	6	(5.1, 7)	7.3	(6.3, 8.4)	5	(4.1, 6)	3.8	(2.9, 4.9)	2.6	(2, 3.4)
10th Grade	14.2	(11.1, 18)	10.9	(9.7, 12.3)	9.2	(7.8, 10.9)	7.4	(6.1, 9)	8.9	(7.6, 10.4)	7.9	(6.6, 9.3)	8.3	(6.8, 10)	8	(6.8, 9.4)	6.5	(5.2, 8)	4.6	(3.6, 5.8)	4.1	(3.3, 5)
11th Grade	12.3	(11.1, 13.6)	11.5	(9.6, 13.7)	9.3	(8, 10.9)	7.5	(6.4, 8.9)	9.6	(8.2, 11.2)	7.6	(6.5, 8.9)	9.7	(8.3, 11.2)	7.6	(6.1, 9.4)	9.2	(7.6, 11.1)	7.2	(6.1, 8.6)	4.8	(3.7, 6.3)
12th Grade	13.9	(11.2, 17.3)	14	(11.5, 16.8)	9	(7.7, 10.5)	8	(6.7, 9.6)	8.9	(7.7, 10.4)	9.1	(7.6, 10.7)	9.1	(8, 10.3)	9.6	(8.3, 11.1)	6.6	(5.5, 7.9)	6.2	(5, 7.7)	5.8	(4.8, 7.2)
Race/Ethnicity																						
NH White	13	(11.6, 14.5)	11.9	(10.9, 13)	9	(8.2, 9.8)	7.4	(6.5, 8.5)	9.3	(8.4, 10.4)	8	(7.1, 9)	8.5	(7.4, 9.6)	8.3	(7.3, 9.4)	7.1	(6.2, 8.3)	6	(4.9, 7.4)	4.9	(4.2, 5.8)
NH Black	11.5	(9.2, 14.2)	9.2	(7.5, 11.2)	7.7	(6.4, 9.3)	6.1	(5, 7.5)	6.7	(5.5, 8.1)	5.1	(4, 6.4)	4.5	(3.7, 5.4)	5.4	(4.3, 6.9)	3.8	(2.5, 5.5)	3.5	(2.6, 4.8)	2.6	(1.9, 3.7)
Hispanic	16.6	(14.6, 18.8)	14.8	(12.7, 17.2)	12.8	(10.6, 15.3)	8.8	(7.5, 10.5)	10.6	(8.8, 12.8)	8.7	(6.9, 10.9)	10.2	(8.9, 11.6)	9.5	(7.8, 11.5)	7.9	(6.1, 10.1)	5.5	(4.6, 6.5)	4.2	(3.5, 5.1)
NH Asian	8.3	(5.6, 12.1)	8.4	(5.3, 13.1)	6.6	(4.6, 9.5)	5	(3.2, 7.8)	3.9	(2.4, 6.3)	3.9	(2.3, 6.3)	2.6	(1.5, 4.6)	4.5	(2.5, 8)	4.4	(3, 6.4)	3.2	(1.7, 5.9)	1	(0.4, 2.6)
NH Other	13.4	(7.3, 23.2)	13.1	(9.4, 17.9)	13.2	(10.4, 16.6)	6.5	(4.5, 9.4)	8.7	(6, 12.4)	12.1	(7.2, 19.6)	10.3	(7.7, 13.8)	9.5	(6.7, 13.3)	7.2	(5.1, 10.1)	6	(3.7, 9.7)	3.9	(2.6, 5.7)
Infrequent Smoking (6-9 days)	2.6	(2.2, 3)	2.9	(2.4, 3.4)	2.4	(2.1, 2.7)	2.1	(1.9, 2.4)	2	(1.7, 2.2)	1.9	(1.5, 2.4)	1.8	(1.5, 2)	1.7	(1.4, 2)	1.7	(1.4, 2.1)	1	(0.8, 1.3)	1	(0.7, 1.4)
Sex																						
Female	2.5	(1.8, 3.5)	3.1	(2.3, 4)	2.3	(1.9, 2.8)	1.8	(1.4, 2.4)	1.6	(1.3, 2.1)	1.7	(1.3, 2.2)	1.7	(1.4, 2)	1.3	(1, 1.6)	1.7	(1.2, 2.2)	1.1	(0.7, 1.5)	0.7	(0.5, 1)
Male	2.6	(2.1, 3.3)	2.7	(2, 3.4)	2.4	(2, 2.9)	2.4	(1.9, 2.9)	2.3	(1.9, 2.7)	2.2	(1.6, 3)	1.9	(1.5, 2.3)	2	(1.6, 2.5)	1.8	(1.4, 2.4)	1	(0.8, 1.2)	1.3	(0.9, 1.8)
Grade																						
9th Grade	3.3	(2.6, 4.1)	2.1	(1.4, 3)	2.3	(1.7, 3)	2.2	(1.5, 3)	2	(1.6, 2.6)	1.4	(1, 2.1)	1.3	(1, 1.8)	1.1	(0.8, 1.6)	1	(0.6, 1.5)	0.8	(0.5, 1.2)	0.8	(0.4, 1.5)
10th Grade	2.8	(1.9, 4.1)	3.6	(2.5, 5.2)	3	(2.2, 4)	1.8	(1.3, 2.5)	1.9	(1.3, 2.6)	2.3	(1.6, 3.4)	1.7	(1.3, 2.3)	1.3	(0.9, 1.8)	1.6	(1.1, 2.2)	0.6	(0.3, 1)	1	(0.6, 1.5)
11th Grade	2.1	(1.6, 2.8)	3.2	(2.3, 4.3)	2.3	(1.8, 2.8)	2.2	(1.7, 2.8)	1.9	(1.5, 2.4)	1.8	(1.3, 2.5)	1.8	(1.4, 2.4)	2.1	(1.7, 2.7)	2.2	(1.3, 3.5)	1.3	(0.9, 2)	0.8	(0.4, 1.4)
12th Grade	2.2	(1.5, 3.3)	2.8	(1.6, 4.7)	1.7	(1.3, 2.4)	2.3	(1.8, 2.9)	2	(1.4, 2.9)	2.3	(1.5, 3.5)	2.3	(1.8, 2.9)	2.2	(1.7, 2.7)	2.3	(1.8, 3.1)	1.5	(1, 2.1)	1.5	(0.9, 2.3)
Race/Ethnicity																						
NH White	2.6	(2.2, 3.1)	2.9	(2.3, 3.7)	2.6	(2.2, 3)	2.3	(2, 2.6)	2	(1.7, 2.4)	2.3	(1.8, 3)	1.9	(1.6, 2.3)	1.8	(1.4, 2.2)	1.9	(1.4, 2.6)	1.2	(0.9, 1.7)	1.4	(1, 1.9)
NH Black	2.2	(1.4, 3.3)	2.2	(1, 4.6)	1.1	(0.6, 1.8)	1.8	(1.3, 2.6)	1.1	(0.8, 1.5)	1.1	(0.7, 1.7)	1.2	(0.8, 1.8)	1.2	(0.8, 2)	0.7	(0.4, 1.3)	0.5	(0.2, 1.1)	0.3	(0.1, 0.7)
Hispanic	1.9	(1.3, 2.7)	2.8	(1.8, 4.3)	3.2	(2.4, 4.4)	2.2	(1.8, 2.7)	2.7	(1.9, 3.8)	1.6	(1.2, 2.2)	1.7	(1.3, 2.3)	1.8	(1.4, 2.4)	1.9	(1.3, 2.8)	0.8	(0.6, 1.2)	0.5	(0.3, 0.9)
NH Asian	2.5	(1.5, 4.1)	3.3	(1.7, 6)	0.9	(0.3, 2.7)	1.7	(0.6, 4.6)	0.9	(0.2, 3.5)	0.7	(0.2, 3.4)	0.7	(0.3, 1.5)	0.4	(0.1, 1)	2.3	(0.8, 6.6)	0.7	(0.1, 3.2)	0.4	(0.1, 1.8)
NH Other	7.4	(2.9, 17.5)	3.7	(2.2, 6)	1.9	(1, 3.6)	0.8	(0.2, 2.5)	1.9	(0.9, 4.2)	1.5	(0.9, 2.4)	2.4	(1.4, 4.2)	1.7	(0.8, 3.5)	1.2	(0.5, 3)	1.2	(0.5, 2.6)	1.3	(0.6, 2.7)
Moderate Smoking (10-19 days)	3.6	(3.1, 4.2)	3.3	(2.7, 4)	2.9	(2.5, 3.4)	2.8	(2.3, 3.3)	2.8	(2.4, 3.3)	2.3	(1.9, 2.7)	2.3	(2, 2.6)	1.9	(1.6, 2.2)	1.6	(1.3, 2)	1	(0.8, 1.3)	0.9	(0.7, 1.1)
Sex																						
Female	3.9	(3.1, 4.8)	3.8	(3, 4.9)	2.8	(2.2, 3.6)	2.7	(2.1, 3.5)	2.9	(2.3, 3.7)	2.1	(1.7, 2.7)	2.2	(1.8, 2.7)	1.6	(1.2, 2.1)	1.4	(1, 2)	0.8	(0.6, 1.2)	0.8	(0.5, 1.1)
Male	3.4	(2.7, 4.3)	2.8	(2.2, 3.7)	3	(2.4, 3.8)	2.8	(2.4, 3.4)	2.7	(2.2, 3.2)	2.4	(1.9, 3)	2.3	(2, 2.7)	2.1	(1.7, 2.7)	1.8	(1.5, 2.1)	1.2	(0.8, 1.7)	1	(0.7, 1.4)

	1997		1999		2001		2003		2005		2007		2009		2011		2013		2015		2017	
	%	(95% CI)	%	(95% CI)	%	(95% CI)	%	(95% CI)	%	(95% CI)	%	(95% CI)	%	(95% CI)	%	(95% CI)	%	(95% CI)	%	(95% CI)	%	(95% CI)
Grade																						
9th Grade	3.9	(3.1, 4.8)	2.8	(2.1, 3.6)	2.9	(2.1, 4.1)	2.2	(1.6, 3)	2.5	(1.9, 3.3)	1.9	(1.3, 2.7)	1.6	(1.1, 2.3)	1.3	(0.9, 1.8)	1.4	(0.9, 2)	0.8	(0.4, 1.5)	0.5	(0.3, 0.8)
10th Grade	3.4	(2.8, 4.2)	5	(3.4, 7.3)	2.4	(1.8, 3.3)	3.4	(2.2, 5)	3	(2, 4.4)	2.4	(1.8, 3.1)	2.6	(1.8, 3.6)	2	(1.5, 2.6)	1.1	(0.7, 1.7)	0.7	(0.4, 1.2)	0.7	(0.5, 1.2)
11th Grade	3.3	(2.3, 4.8)	2.7	(1.9, 3.8)	3	(2.3, 3.9)	2.7	(1.9, 3.8)	2.5	(1.8, 3.3)	2	(1.4, 2.9)	2.5	(1.9, 3.3)	1.8	(1.4, 2.4)	2.1	(1.6, 2.9)	1.3	(0.8, 1.9)	1.1	(0.7, 1.7)
12th Grade	4.1	(2.9, 5.6)	2.9	(2.2, 3.8)	3.5	(2.6, 4.5)	2.9	(2, 4.1)	3.4	(2.7, 4.2)	2.9	(2.1, 3.9)	2.6	(2, 3.3)	2.5	(2, 3.2)	1.9	(1.3, 2.7)	1.3	(0.8, 2.2)	1.3	(0.9, 2)
Race/Ethnicity																						
NH White	4.2	(3.6, 5)	3.7	(3, 4.5)	3.2	(2.7, 3.9)	3.4	(2.8, 4.1)	3.4	(2.7, 4.2)	2.5	(2.1, 2.9)	2.6	(2.2, 3.1)	2.1	(1.7, 2.7)	1.9	(1.5, 2.4)	1.2	(0.8, 1.8)	1.2	(0.9, 1.6)
NH Black	1.9	(1.3, 2.6)	1.3	(0.8, 2.2)	1.3	(0.8, 2.2)	1.6	(1, 2.6)	1.4	(0.9, 2.1)	1.6	(1.2, 2.2)	1.7	(1, 2.9)	1.2	(0.7, 1.9)	1	(0.4, 2.4)	0.7	(0.4, 1.3)	0.4	(0.2, 0.7)
Hispanic	4.7	(3.6, 6.1)	4.7	(3.3, 6.7)	3.3	(2.4, 4.6)	1.9	(1.4, 2.6)	2.3	(1.7, 3.1)	2.1	(1.5, 3)	1.9	(1.4, 2.6)	1.7	(1.3, 2.4)	1.3	(0.9, 2)	0.8	(0.5, 1.3)	0.6	(0.4, 1.1)
NH Asian	2.2	(1.4, 3.5)	1	(0.5, 2.2)	0.9	(0.1, 5.7)	2.4	(1.2, 4.7)	0.9	(0.2, 4)	1.1	(0.3, 3.3)	1	(0.4, 2.4)	####	#VALUE!	0.9	(0.4, 2.1)	0.1	(0, 0.4)	0.5	(0.2, 1.2)
NH Other	2.5	(1.1, 5.7)	3.1	(1.3, 6.9)	3.7	(1.3, 9.8)	1.5	(0.7, 2.9)	1.7	(1, 2.8)	2.5	(1, 6.3)	2.5	(1.6, 3.7)	2.4	(1.5, 3.6)	1.4	(0.6, 3)	1.3	(0.8, 2.1)	0.3	(0.1, 1)
Frequent Smoking (20-29 days)	4.5	(3.8, 5.4)	3.9	(3.3, 4.6)	3.6	(3.1, 4.1)	2.1	(1.8, 2.5)	2.2	(1.8, 2.7)	1.9	(1.6, 2.4)	2	(1.7, 2.2)	1.6	(1.4, 1.9)	1.6	(1.3, 2)	1.1	(0.8, 1.4)	0.6	(0.4, 0.9)
Sex																						
Female	4.4	(3.5, 5.6)	3.9	(3.1, 4.8)	3.3	(2.7, 3.9)	2.3	(1.8, 2.9)	2.4	(1.8, 3.1)	1.6	(1.3, 2.1)	1.8	(1.5, 2.3)	1.2	(1, 1.5)	1.6	(1.2, 2.2)	1.1	(0.7, 1.6)	0.5	(0.3, 0.9)
Male	4.6	(3.9, 5.4)	3.9	(3.1, 4.9)	3.9	(3.3, 4.7)	2	(1.7, 2.5)	2	(1.6, 2.5)	2.2	(1.8, 2.9)	2.1	(1.8, 2.4)	2.1	(1.7, 2.5)	1.6	(1.2, 2.1)	1.1	(0.7, 1.6)	0.7	(0.4, 1)
Grade																						
9th Grade	3.7	(2.5, 5.5)	2.7	(1.9, 3.9)	2.8	(1.9, 4)	1.3	(0.9, 1.8)	1.7	(1.1, 2.7)	1.1	(0.8, 1.6)	1.3	(0.9, 1.9)	1	(0.7, 1.6)	0.7	(0.4, 1)	0.5	(0.3, 0.9)	0.4	(0.1, 1.1)
10th Grade	4.8	(3.3, 6.9)	4.3	(3.1, 5.9)	3.1	(2.4, 4.1)	2.2	(1.7, 2.8)	1.9	(1.4, 2.6)	1.5	(0.9, 2.4)	1.7	(1.3, 2.2)	0.9	(0.6, 1.4)	1.1	(0.7, 1.7)	0.8	(0.5, 1.4)	0.4	(0.2, 0.7)
11th Grade	4.8	(4, 5.8)	4.2	(2.9, 6.1)	3	(2.4, 3.8)	2.3	(1.6, 3.2)	2.3	(1.6, 3.4)	3.2	(2.5, 4.1)	2.2	(1.7, 3)	2.1	(1.7, 2.7)	2.5	(1.8, 3.4)	1.3	(0.8, 2.2)	0.6	(0.3, 1)
12th Grade	4.7	(3.7, 5.9)	4.6	(3.7, 5.7)	5.8	(4.8, 7.1)	3.1	(2.4, 4)	3.1	(2.3, 4.1)	2.3	(1.8, 2.9)	2.8	(2.1, 3.7)	2.6	(2.1, 3.3)	2.3	(1.7, 3.1)	1.8	(1.2, 2.6)	1.2	(0.8, 1.7)
Race/Ethnicity																						
NH White	5.5	(4.6, 6.4)	4.7	(3.8, 5.9)	4.3	(3.7, 4.9)	2.6	(2.2, 3.1)	2.5	(2, 3.2)	2.3	(1.8, 2.9)	2.6	(2.2, 3)	2	(1.6, 2.5)	2.1	(1.6, 2.7)	1.2	(0.9, 1.7)	0.9	(0.7, 1.3)
NH Black	1.7	(1.3, 2.3)	0.9	(0.6, 1.4)	1.1	(0.7, 1.8)	1.2	(0.8, 1.8)	0.7	(0.4, 1.1)	1.4	(0.9, 2.2)	0.7	(0.3, 1.3)	0.5	(0.2, 0.9)	1	(0.6, 1.7)	0.3	(0.1, 0.7)	0	(0, 0.1)
Hispanic	3.6	(2.4, 5.2)	3.1	(2.4, 4.1)	2.6	(1.8, 3.7)	1.6	(1.1, 2.3)	1.8	(1.1, 2.7)	1.3	(0.9, 2)	1.2	(0.9, 1.7)	1.3	(0.9, 1.9)	1	(0.7, 1.6)	0.6	(0.3, 0.9)	0.4	(0.2, 0.8)
NH Asian	2.9	(1.7, 4.9)	1.1	(0.6, 1.9)	2.6	(1.1, 5.7)	1.4	(0.6, 3.1)	1	(0.5, 2.4)	1.5	(0.7, 3)	1	(0.5, 1.9)	0.5	(0.1, 2.3)	1.2	(0.3, 4.4)	1.7	(0.5, 5.2)	.	.
NH Other	5.1	(1.2, 19.5)	4.3	(2.6, 7)	4.2	(2.6, 6.5)	1.5	(0.8, 3)	4.3	(2.5, 7.1)	2.1	(1.1, 3.9)	2	(1, 4)	2	(1.1, 3.4)	0.9	(0.4, 2)	1.9	(0.8, 4.6)	0.2	(0, 1.3)
Daily Smoking (all 30 days)	12.2	(10.6, 14)	12.8	(10.7, 15.3)	10.3	(9, 11.6)	7.5	(6.4, 8.8)	7.1	(6, 8.4)	6.1	(5, 7.5)	5.3	(4.6, 6.1)	4.7	(4.1, 5.4)	4	(3.1, 5.2)	2.3	(1.8, 3)	2	(1.4, 2.8)
Sex																						
Female	11.3	(9.6, 13.2)	11.8	(9.9, 13.9)	9.6	(8.3, 11.1)	7.4	(6, 9.1)	6.9	(5.7, 8.4)	5.8	(4.5, 7.4)	4.6	(3.7, 5.6)	4.2	(3.3, 5.3)	3.8	(2.6, 5.6)	2.2	(1.6, 2.9)	2	(1.3, 3.1)
Male	12.9	(10.6, 15.7)	13.9	(11.1, 17.3)	10.9	(9.5, 12.5)	7.6	(6.4, 9)	7.3	(6.2, 8.6)	6.5	(5.3, 7.9)	6	(5.2, 6.7)	5.3	(4.7, 6)	4.2	(3.4, 5.4)	2.4	(1.8, 3.2)	2	(1.5, 2.7)
Grade																						
9th Grade	9.4	(6.6, 13.2)	8.5	(6.9, 10.4)	6.2	(4.6, 8.3)	5	(3.6, 6.7)	5.2	(3.9, 6.7)	3.2	(2.3, 4.4)	3.3	(2.6, 4.2)	2.3	(1.7, 3)	2.2	(1.5, 3.2)	1.7	(1, 3)	0.9	(0.6, 1.6)
10th Grade	10.2	(8.7, 11.9)	10.9	(8.3, 14.2)	9.2	(7.9, 10.7)	7.1	(5.6, 8.9)	5.8	(4.4, 7.5)	5.5	(4.2, 7.2)	4	(3.1, 5.1)	3.4	(2.6, 4.4)	2.9	(2, 4.2)	2.1	(1.4, 3)	1.4	(0.9, 2.3)
11th Grade	14.1	(11.5, 17.1)	14.5	(11.6, 17.9)	12.2	(10, 14.7)	8.9	(7.1, 11.2)	8	(6.4, 10)	6.9	(4.9, 9.7)	6.1	(5.1, 7.2)	5.6	(4.5, 6.8)	5.1	(3.6, 7.3)	1.9	(1.3, 2.7)	2.2	(1.4, 3.6)
12th Grade	14.7	(12, 17.8)	18.5	(12.9, 25.9)	15.1	(12.4, 18.3)	10	(8, 12.3)	10.1	(8.2, 12.4)	9.9	(7.6, 12.8)	8.4	(7.1, 10)	8.2	(7, 9.6)	6.1	(4.7, 7.9)	3.4	(2.4, 4.7)	3.5	(2.4, 5)
Race/Ethnicity																						
NH White	14.4	(12.6, 16.5)	15.5	(13.3, 17.9)	12.9	(11.4, 14.6)	9.2	(7.9, 10.7)	8.6	(7.3, 10.2)	8.1	(6.6, 10)	6.9	(5.9, 8.2)	6	(5.2, 7)	5.6	(4.2, 7.4)	2.8	(1.9, 3.9)	2.6	(1.8, 3.7)
NH Black	5.5	(4.1, 7.3)	6.1	(3.3, 10.8)	3.5	(2.3, 5.3)	4.3	(3.1, 5.9)	3.1	(1.9, 4.9)	2.5	(1.7, 3.6)	1.5	(1, 2.2)	2.2	(1.5, 3.1)	1.7	(1.1, 2.8)	1.4	(0.8, 2.5)	1.1	(0.5, 2.2)
Hispanic	7.3	(5.2, 10.1)	7.3	(5, 10.7)	4.7	(3.6, 6.1)	3.9	(3.1, 5)	4.7	(3.4, 6.5)	2.9	(2.1, 3.9)	3	(2.4, 3.8)	3.1	(2.6, 3.7)	1.9	(1.4, 2.5)	1.6	(1.1, 2.2)	1.3	(0.9, 1.9)
NH Asian	5.2	(3.1, 8.4)	9.1	(4.9, 16.1)	4.2	(2.1, 8.3)	2.6	(1.1, 5.7)	3.2	(1.4, 6.9)	2.8	(1.2, 6.4)	2.2	(1.2, 4.2)	2.2	(1, 4.7)	1.6	(0.5, 5.1)	1.4	(0.5, 3.5)	0.3	(0.1, 1.1)
NH Other	16	(9.1, 26.6)	11.4	(7.6, 16.9)	9	(6.3, 12.7)	10.4	(6.7, 15.7)	9.4	(6.2, 14)	5.3	(3.8, 7.4)	5.5	(3.4, 8.7)	5.5	(3.9, 7.8)	3.6	(1.9, 6.7)	3.3	(1.9, 5.6)	2.2	(1.1, 4.3)

Table S3. Average cigarettes smoked per day among current, rare, infrequent, moderate, frequent, and daily smoking high school students, by sex, grade, and race/ethnicity, National Youth Risk Behavior Survey 1997-2017

	1997		1999		2001		2003		2005		2007		2009		2011		2013		2015		2017	
	%	(95% CI)	%	(95% CI)	%	(95% CI)	%	(95% CI)	%	(95% CI)	%	(95% CI)	%	(95% CI)	%	(95% CI)	%	(95% CI)	%	(95% CI)	%	(95% CI)
Current Smoking	4.1	(3.7, 4.5)	4.3	(3.8, 4.8)	4.3	(3.9, 4.7)	4.1	(3.7, 4.4)	3.5	(3.1, 3.8)	3.5	(3.1, 3.9)	3	(2.8, 3.2)	2.9	(2.5, 3.2)	2.9	(2.5, 3.4)	2.7	(2.3, 3.1)	2.9	(2.5, 3.4)
Sex																						
Female	3.6	(3.2, 4)	3.8	(3.3, 4.2)	3.8	(3.3, 4.2)	3.6	(3.2, 4)	3	(2.6, 3.4)	3	(2.6, 3.5)	2.4	(2.1, 2.7)	2.5	(2, 2.9)	2.5	(1.8, 3.2)	2.5	(1.9, 3)	2.7	(2.1, 3.3)
Male	4.5	(3.9, 5)	4.8	(4.2, 5.4)	4.8	(4.4, 5.3)	4.4	(3.9, 5)	3.9	(3.5, 4.2)	3.9	(3.5, 4.4)	3.6	(3.2, 3.9)	3.1	(2.8, 3.5)	3.3	(2.9, 3.7)	2.8	(2.3, 3.4)	3	(2.5, 3.4)
Grade																						
9th Grade	3.6	(2.8, 4.4)	3.6	(3, 4.2)	3.2	(2.5, 3.8)	3.4	(2.6, 4.3)	2.9	(2.3, 3.4)	2.8	(2.2, 3.5)	2.9	(2.3, 3.4)	2.2	(1.7, 2.7)	2.7	(2, 3.5)	2.5	(1.7, 3.3)	2.2	(1.6, 2.8)
10th Grade	3.5	(2.8, 4.1)	3.7	(3.1, 4.3)	4	(3.5, 4.5)	3.5	(2.9, 4.2)	2.8	(2.3, 3.2)	3.2	(2.6, 3.7)	2.6	(2.2, 2.9)	2.4	(1.8, 3)	2.7	(2, 3.4)	3	(2.2, 3.8)	2.7	(1.7, 3.7)
11th Grade	4.4	(4, 4.9)	4.3	(3.7, 4.9)	4.6	(4, 5.1)	4.2	(3.6, 4.7)	3.8	(3.2, 4.3)	3.5	(2.8, 4.2)	3	(2.7, 3.3)	3.1	(2.6, 3.6)	2.6	(2.1, 3.2)	1.9	(1.4, 2.4)	2.5	(1.7, 3.3)
12th Grade	4.6	(3.6, 5.6)	5.3	(4, 6.6)	5.3	(4.6, 6)	4.8	(4.1, 5.5)	4.2	(3.7, 4.6)	4.3	(3.5, 5)	3.5	(3.1, 3.8)	3.3	(2.8, 3.8)	3.5	(2.6, 4.3)	3	(2.3, 3.7)	3.4	(2.6, 4.1)
Race/Ethnicity																						
NH White	4.5	(4, 4.9)	4.8	(4.2, 5.3)	4.8	(4.3, 5.3)	4.1	(3.7, 4.6)	3.8	(3.4, 4.1)	3.9	(3.4, 4.3)	3.2	(3, 3.5)	3.1	(2.7, 3.5)	3.4	(2.9, 4)	2.5	(2.1, 2.9)	2.8	(2.3, 3.3)
NH Black	2.3	(1.8, 2.8)	2.4	(1.9, 2.9)	2.6	(1.9, 3.3)	3	(2.2, 3.8)	2	(1.3, 2.6)	2.5	(1.9, 3.1)	2.2	(1.3, 3.1)	2.2	(1.5, 3)	1.9	(1.3, 2.6)	2.5	(1.4, 3.6)	3.3	(1.7, 4.9)
Hispanic	2.5	(1.9, 3.1)	2.7	(2.1, 3.3)	2.4	(1.8, 2.9)	2.8	(2.2, 3.3)	2.5	(1.8, 3.2)	2.4	(1.9, 3)	2.1	(1.7, 2.4)	2.2	(1.7, 2.7)	1.9	(1.5, 2.3)	2.5	(1.6, 3.4)	2.4	(1.6, 3.3)
NH Asian	3.1	(2.2, 3.9)	3.2	(2.1, 4.4)	3.5	(1.9, 5.1)	4	(1.8, 6.1)	2.7	(1, 4.5)	3.8	(2.3, 5.4)	5.2	(2.5, 7.9)	3.5	(0.9, 6.1)	3.4	(0, 6.8)	4.9	(1.7, 8.1)	1.7	(0, 3.3)
NH Other	5.1	(3, 7.1)	3.9	(2.5, 5.2)	3.2	(2.3, 4.2)	8.2	(5.9, 10.5)	4	(3.1, 5)	2.4	(1.5, 3.4)	2.9	(1.9, 3.8)	3.2	(2.2, 4.1)	1.7	(1.1, 2.3)	3.9	(2.1, 5.7)	3.6	(1.7, 5.5)
Rare Smoking (1-5 days)	0.2	(0.1, 0.2)	0.1	(0.1, 0.2)	0.1	(0.1, 0.2)	0.2	(0.1, 0.2)	0.1	(0.1, 0.2)	0.2	(0.1, 0.2)	0.2	(0.1, 0.2)	0.1	(0.1, 0.2)	0.2	(0.1, 0.2)	0.1	(0.1, 0.1)	0.1	(0.1, 0.2)
Sex																						
Female	0.2	(0.1, 0.2)	0.1	(0.1, 0.1)	0.1	(0.1, 0.1)	0.2	(0.1, 0.2)	0.1	(0.1, 0.2)	0.2	(0.1, 0.2)	0.2	(0.1, 0.2)	0.1	(0.1, 0.2)	0.1	(0.1, 0.2)	0.1	(0.1, 0.1)	0.1	(0.1, 0.1)
Male	0.2	(0.1, 0.2)	0.1	(0.1, 0.2)	0.2	(0.2, 0.2)	0.2	(0.1, 0.2)	0.1	(0.1, 0.2)	0.2	(0.1, 0.2)	0.2	(0.1, 0.2)	0.1	(0.1, 0.2)	0.2	(0.1, 0.2)	0.1	(0.1, 0.2)	0.1	(0.1, 0.2)
Grade																						
9th Grade	0.1	(0.1, 0.2)	0.1	(0.1, 0.2)	0.1	(0.1, 0.2)	0.1	(0.1, 0.2)	0.1	(0.1, 0.1)	0.1	(0.1, 0.2)	0.1	(0.1, 0.2)	0.1	(0.1, 0.2)	0.1	(0.1, 0.1)	0.1	(0.1, 0.1)	0.1	(0.1, 0.2)
10th Grade	0.2	(0.1, 0.2)	0.1	(0.1, 0.2)	0.1	(0.1, 0.2)	0.2	(0.1, 0.2)	0.2	(0.1, 0.2)	0.2	(0.1, 0.2)	0.1	(0.1, 0.2)	0.2	(0.1, 0.2)	0.2	(0.1, 0.2)	0.1	(0.1, 0.2)	0.1	(0.1, 0.2)
11th Grade	0.2	(0.1, 0.2)	0.1	(0.1, 0.1)	0.1	(0.1, 0.2)	0.2	(0.1, 0.2)	0.2	(0.1, 0.2)	0.2	(0.1, 0.2)	0.2	(0.1, 0.2)	0.1	(0.1, 0.2)	0.2	(0.1, 0.2)	0.1	(0.1, 0.2)	0.1	(0.1, 0.2)
12th Grade	0.2	(0.1, 0.2)	0.2	(0.1, 0.2)	0.2	(0.1, 0.2)	0.1	(0.1, 0.2)	0.2	(0.1, 0.2)	0.2	(0.1, 0.2)	0.2	(0.1, 0.2)	0.1	(0.1, 0.2)	0.1	(0.1, 0.2)	0.1	(0.1, 0.2)	0.1	(0.1, 0.2)
Race/Ethnicity																						
NH White	0.2	(0.1, 0.2)	0.1	(0.1, 0.2)	0.2	(0.1, 0.2)	0.2	(0.1, 0.2)	0.1	(0.1, 0.2)	0.2	(0.1, 0.2)	0.2	(0.1, 0.2)	0.1	(0.1, 0.2)	0.2	(0.1, 0.2)	0.1	(0.1, 0.1)	0.1	(0.1, 0.2)
NH Black	0.1	(0.1, 0.2)	0.1	(0.1, 0.1)	0.1	(0.1, 0.2)	0.1	(0.1, 0.2)	0.1	(0.1, 0.1)	0.1	(0.1, 0.2)	0.1	(0.1, 0.2)	0.1	(0.1, 0.1)	0.1	(0.1, 0.2)	0.1	(0.1, 0.2)	0.1	(0.1, 0.2)
Hispanic	0.2	(0.1, 0.2)	0.1	(0.1, 0.2)	0.1	(0.1, 0.2)	0.1	(0.1, 0.2)	0.1	(0.1, 0.2)	0.2	(0.1, 0.2)	0.1	(0.1, 0.1)	0.2	(0.1, 0.2)	0.1	(0.1, 0.1)	0.1	(0.1, 0.1)	0.1	(0.1, 0.2)
NH Asian	0.1	(0.1, 0.2)	0.2	(0.1, 0.2)	0.2	(0.1, 0.2)	0.2	(0.1, 0.3)	0.1	(0, 0.2)	0.1	(0.1, 0.1)	0.1	(0, 0.1)	0.2	(0.1, 0.4)	0.2	(0.1, 0.3)	0.1	(0, 0.1)	0.1	(0.1, 0.1)
NH Other	0.2	(0.1, 0.4)	0.1	(0.1, 0.2)	0.1	(0.1, 0.2)	0.1	(0.1, 0.2)	0.2	(0.1, 0.2)	0.2	(0.1, 0.3)	0.2	(0.1, 0.2)	0.1	(0.1, 0.2)	0.2	(0.1, 0.2)	0.2	(0.1, 0.3)	0.2	(0.1, 0.3)
Infrequent Smoking (6-9 days)	0.8	(0.7, 1)	0.7	(0.7, 0.7)	0.7	(0.6, 0.8)	0.7	(0.6, 0.8)	0.7	(0.6, 0.8)	0.7	(0.6, 0.8)	0.7	(0.6, 0.8)	0.7	(0.6, 0.8)	0.7	(0.6, 0.8)	0.7	(0.6, 0.7)	0.8	(0.7, 1)
Sex																						
Female	0.7	(0.6, 0.9)	0.7	(0.7, 0.8)	0.6	(0.5, 0.7)	0.7	(0.6, 0.8)	0.7	(0.6, 0.8)	0.7	(0.6, 0.8)	0.7	(0.6, 0.8)	0.6	(0.5, 0.8)	0.6	(0.5, 0.8)	0.6	(0.5, 0.7)	0.7	(0.5, 0.9)
Male	0.9	(0.7, 1.2)	0.7	(0.6, 0.8)	0.7	(0.6, 0.9)	0.7	(0.6, 0.8)	0.7	(0.5, 0.9)	0.7	(0.6, 0.9)	0.7	(0.6, 0.9)	0.7	(0.6, 0.8)	0.8	(0.7, 0.9)	0.7	(0.6, 0.9)	0.9	(0.7, 1.1)
Grade																						
9th Grade	0.7	(0.5, 0.8)	0.6	(0.5, 0.8)	0.6	(0.5, 0.7)	0.6	(0.5, 0.8)	0.6	(0.5, 0.7)	0.8	(0.4, 1.1)	0.6	(0.5, 0.7)	0.6	(0.5, 0.8)	0.6	(0.4, 0.7)	0.5	(0.4, 0.6)	1.3	(0.8, 1.8)
10th Grade	1	(0.7, 1.4)	0.7	(0.7, 0.8)	0.7	(0.6, 0.9)	0.7	(0.6, 0.8)	0.7	(0.5, 0.9)	0.8	(0.7, 1)	0.8	(0.6, 0.9)	0.7	(0.6, 0.8)	0.8	(0.6, 1)	0.7	(0.5, 0.8)	0.8	(0.4, 1.1)
11th Grade	1	(0.5, 1.4)	0.7	(0.6, 0.8)	0.7	(0.6, 0.8)	0.7	(0.6, 0.8)	0.9	(0.5, 1.3)	0.6	(0.5, 0.7)	0.8	(0.6, 0.9)	0.8	(0.6, 1)	0.6	(0.5, 0.8)	0.8	(0.6, 1)	0.6	(0.4, 0.7)
12th Grade	0.8	(0.7, 0.8)	0.7	(0.5, 0.8)	0.7	(0.5, 0.8)	0.8	(0.6, 0.9)	0.6	(0.5, 0.8)	0.6	(0.5, 0.7)	0.7	(0.6, 0.8)	0.6	(0.5, 0.8)	0.8	(0.6, 0.9)	0.6	(0.5, 0.7)	0.7	(0.5, 0.8)
Race/Ethnicity																						
NH White	0.9	(0.6, 1.1)	0.7	(0.6, 0.7)	0.7	(0.6, 0.8)	0.7	(0.6, 0.7)	0.7	(0.6, 0.9)	0.7	(0.6, 0.9)	0.7	(0.6, 0.9)	0.7	(0.6, 0.8)	0.7	(0.5, 0.8)	0.7	(0.5, 0.8)	0.8	(0.6, 1)
NH Black	0.9	(0.5, 1.2)	0.9	(0.7, 1)	0.6	(0.4, 0.8)	0.7	(0.5, 0.8)	0.7	(0.4, 1.1)	0.7	(0.5, 0.8)	0.6	(0.4, 0.8)	1	(0.7, 1.3)	0.6	(0.4, 0.7)	0.4	(0.2, 0.5)	2.3	(0.8, 3.9)
Hispanic	0.7	(0.5, 0.8)	0.6	(0.5, 0.7)	0.6	(0.5, 0.7)	0.7	(0.5, 0.8)	0.6	(0.5, 0.7)	0.6	(0.5, 0.7)	0.6	(0.5, 0.8)	0.5	(0.4, 0.6)	0.8	(0.6, 1.1)	0.7	(0.5, 0.9)	0.8	(0.3, 1.3)
NH Asian	0.8	(0.7, 0.9)	0.5	(0.4, 0.7)	0.6	(0.2, 1)	1.7	(1.5, 1.9)	0.9	(0.9, 0.9)	0.9	(0.8, 1)	0.6	(0.2, 1)	0.9	(0.9, 0.9)	0.9	(0.5, 1.2)	0.8	(0.3, 1.2)	1.6	(-0.8, 3.9)
NH Other	0.4	(0.2, 0.5)	0.7	(0.5, 0.9)	0.6	(0.4, 0.8)	0.6	(0.2, 0.9)	0.6	(0.4, 0.8)	0.6	(0.4, 0.8)	1	(0.8, 1.2)	0.9	(0.8, 1)	0.6	(0.3, 0.9)	0.5	(0.2, 0.8)	0.8	(0.5, 1.1)
Moderate Smoking (10-19 days)	1.8	(1.6, 2.1)	1.7	(1.5, 1.8)	1.8	(1.7, 2)	1.7	(1.5, 2)	1.7	(1.5, 2)	1.8	(1.6, 2.1)	1.6	(1.5, 1.7)	1.5	(1.3, 1.7)	1.9	(1.6, 2.1)	1.8	(1.5, 2.2)	2	(1.5, 2.6)
Sex																						
Female	1.6	(1.4, 1.8)	1.7	(1.5, 1.8)	1.8	(1.6, 1.9)	1.6	(1.3, 2)	1.7	(1.5, 1.9)	1.7	(1.5, 2)	1.7	(1.5, 1.8)	1.5	(1.3, 1.7)	1.7	(1.5, 1.9)	1.5	(1.2, 1.7)	1.9	(1.1, 2.6)
Male	2.1	(1.7, 2.5)	1.7	(1.4, 2)	1.9	(1.6, 2.2)	1.8	(1.5, 2.1)	1.7	(1.4, 2.1)	2	(1.6, 2.3)	1.5	(1.3, 1.7)	1.5	(1.3, 1.8)	2	(1.7, 2.3)	2.1	(1.6, 2.6)	2.2	(1.5, 2.8)

	1997		1999		2001		2003		2005		2007		2009		2011		2013		2015		2017	
	%	(95% CI)	%	(95% CI)	%	(95% CI)	%	(95% CI)	%	(95% CI)	%	(95% CI)	%	(95% CI)	%	(95% CI)	%	(95% CI)	%	(95% CI)	%	(95% CI)
Grade																						
9th Grade	1.5	(1.3, 1.6)	1.8	(1.4, 2.3)	1.7	(1.5, 2)	1.8	(1.3, 2.3)	1.5	(1.1, 1.8)	1.7	(1.3, 2)	1.4	(1.3, 1.6)	1.5	(1.2, 1.8)	2	(1.5, 2.4)	2.2	(1.1, 3.2)	1.6	(1, 2.1)
10th Grade	2	(1.3, 2.6)	1.6	(1.3, 1.8)	2.1	(1.6, 2.5)	1.8	(1.3, 2.4)	1.8	(1.4, 2.1)	2.1	(1.5, 2.7)	1.7	(1.5, 1.9)	1.5	(1.2, 1.7)	1.5	(1.2, 1.7)	1.3	(1, 1.7)	2.6	(1.6, 3.5)
11th Grade	2	(1.5, 2.5)	1.8	(1.6, 2)	2	(1.5, 2.4)	1.7	(1.3, 2.2)	2	(1, 3)	1.7	(1.5, 1.9)	1.6	(1.3, 1.9)	1.3	(1.1, 1.6)	1.9	(1.5, 2.2)	1.8	(1.4, 2.2)	2.1	(1.4, 2.8)
12th Grade	1.8	(1.6, 2)	1.6	(1.1, 2.1)	1.6	(1.4, 1.8)	1.4	(1.2, 1.7)	1.6	(1.4, 1.9)	1.8	(1.4, 2.2)	1.5	(1.2, 1.7)	1.7	(1.4, 2)	1.9	(1.5, 2.3)	1.9	(1.4, 2.5)	1.9	(1, 2.8)
Race/Ethnicity																						
NH White	1.8	(1.6, 2.1)	1.6	(1.5, 1.7)	1.8	(1.6, 2)	1.7	(1.4, 2)	1.8	(1.5, 2.1)	1.9	(1.5, 2.2)	1.6	(1.4, 1.7)	1.5	(1.3, 1.8)	2	(1.7, 2.3)	2	(1.5, 2.5)	2.2	(1.6, 2.8)
NH Black	1.4	(1, 1.8)	2.4	(1.6, 3.3)	2	(1.4, 2.6)	1.4	(1, 1.9)	1.6	(1, 2.3)	1.8	(1.3, 2.3)	1.5	(1.1, 1.9)	1.4	(1.1, 1.6)	1.5	(1.3, 1.8)	1.4	(0.9, 1.8)	1.5	(1, 1.9)
Hispanic	1.8	(1.2, 2.4)	1.7	(1.2, 2.1)	1.8	(1.6, 2)	1.8	(1.2, 2.3)	1.5	(1.1, 1.8)	1.8	(1.4, 2.2)	1.7	(1.5, 2)	1.6	(1.3, 1.8)	1.5	(1.2, 1.8)	1.7	(1.4, 2.1)	1.3	(0.7, 1.9)
NH Asian	1.9	(1.2, 2.6)	1.7	(1.7, 1.7)	1.7	(1.7, 1.7)	2.5	(0.2, 4.8)	1.2	(1.2, 1.2)	2.1	(1.3, 2.9)	1.5	(1.1, 1.8)	.	.	1.4	(0.9, 1.9)	1.7	(1.7, 1.7)	1.5	(0.4, 2.6)
NH Other	2.4	(1.4, 3.5)	1.7	(1.5, 2)	2.2	(1.2, 3.1)	1.7	(1.7, 1.7)	1.2	(0.8, 1.6)	2	(1.4, 2.6)	1.6	(1.2, 2.1)	1.4	(1, 1.8)	1.4	(1.1, 1.7)	1.4	(1.2, 1.7)	1.7	(1.7, 1.7)
Frequent Smoking (20-29 days)	4.1	(3.6, 4.6)	3.9	(3.5, 4.3)	4	(3.6, 4.4)	3.9	(3.5, 4.3)	3.9	(3.5, 4.2)	3.9	(3.4, 4.4)	3.3	(3, 3.6)	3.4	(3.1, 3.7)	3.9	(3.4, 4.4)	4.3	(3.7, 4.9)	3.6	(2.9, 4.3)
Sex																						
Female	3.7	(3.1, 4.2)	3.6	(3.2, 3.9)	3.6	(3, 4.2)	3.7	(3.2, 4.1)	3.4	(2.9, 3.9)	3.2	(2.7, 3.6)	2.9	(2.6, 3.2)	3.4	(2.8, 3.9)	4	(3.1, 4.9)	3.4	(2.7, 4.1)	3.3	(2.6, 3.9)
Male	4.4	(3.9, 4.9)	4.2	(3.6, 4.8)	4.4	(3.9, 4.9)	4.2	(3.6, 4.8)	4.4	(3.8, 5)	4.5	(3.7, 5.2)	3.6	(3.1, 4)	3.4	(3.1, 3.7)	3.7	(3.3, 4.2)	5.1	(4, 6.2)	3.9	(2.9, 4.9)
Grade																						
9th Grade	4.7	(3.7, 5.8)	4.2	(3.3, 5.2)	4.3	(3.4, 5.3)	3.4	(2.6, 4.2)	3.6	(2.6, 4.7)	4.3	(3.2, 5.5)	3	(2.3, 3.6)	3.3	(2.7, 3.9)	3.9	(3, 4.7)	3.2	(1.7, 4.7)	4.6	(1.6, 7.6)
10th Grade	3.7	(3, 4.4)	3.5	(3.1, 3.9)	3.8	(3.3, 4.3)	4.2	(3.2, 5.2)	3.1	(2.5, 3.7)	3.2	(2.7, 3.7)	3.2	(2.8, 3.7)	2.8	(2.3, 3.4)	5	(2.5, 7.5)	4.1	(1.8, 6.4)	3.3	(2.5, 4.1)
11th Grade	4	(2.9, 5)	3.6	(3.1, 4.1)	3.6	(3.1, 4.2)	4.3	(3.4, 5.2)	4.6	(3.5, 5.6)	4	(2.9, 5.2)	3.2	(2.8, 3.6)	3.6	(3.1, 4.2)	3.8	(2.7, 4.8)	4.5	(3.6, 5.4)	4.2	(3.1, 5.2)
12th Grade	4.1	(3, 5.2)	4.3	(3.4, 5.2)	4.1	(3.4, 4.8)	3.6	(2.9, 4.3)	4	(3.4, 4.5)	4	(3.4, 4.7)	3.6	(3.1, 4)	3.4	(2.9, 4)	3.4	(2.8, 3.9)	4.6	(3.1, 6.1)	3	(2.4, 3.7)
Race/Ethnicity																						
NH White	4	(3.5, 4.5)	3.8	(3.4, 4.3)	4	(3.6, 4.5)	4.1	(3.6, 4.6)	3.9	(3.5, 4.4)	4	(3.4, 4.7)	3.4	(3.1, 3.7)	3.3	(3, 3.6)	4.1	(3.4, 4.8)	4	(3.3, 4.7)	3.7	(2.9, 4.5)
NH Black	3.5	(2.8, 4.2)	3.2	(2.1, 4.3)	3.7	(2.7, 4.7)	2.8	(2.1, 3.4)	2.5	(1.6, 3.4)	3.8	(2.6, 5)	2.6	(1.8, 3.4)	3.2	(2, 4.3)	2.7	(2.1, 3.3)	3.2	(1.2, 5.2)	.	.
Hispanic	4.4	(3, 5.7)	4	(2.8, 5.1)	4.4	(3.5, 5.4)	3.6	(2.6, 4.5)	3.9	(2.8, 5)	3.5	(2.6, 4.3)	3.1	(2.4, 3.7)	4.1	(3, 5.2)	3.7	(2.7, 4.7)	4.1	(2.9, 5.3)	2.4	(1.9, 3)
NH Asian	2.9	(2.8, 3)	4.5	(3.5, 5.5)	3.4	(2.6, 4.3)	3.6	(1.2, 5.9)	2.9	(2.9, 2.9)	3.9	(2.2, 5.7)	2.3	(1.6, 3.1)	1.4	(0.2, 2.5)	2.9	(2.9, 2.9)	8.9	(4.7, 13.1)	.	.
NH Other	3	(2.7, 3.2)	3.8	(3.1, 4.6)	3.7	(2.9, 4.4)	4.2	(2.8, 5.7)	3.8	(2.6, 5.1)	3.2	(1.3, 5.1)	2.5	(2.1, 2.9)	3.7	(2.1, 5.4)	3.2	(2.6, 3.8)	3.6	(2.4, 4.8)	5.7	(3.7, 7.7)
Daily Smoking (all 30 days)	9.8	(9.2, 10.4)	9.8	(9.2, 10.3)	9.7	(9.3, 10.1)	9.6	(9.2, 10.1)	8.8	(8.3, 9.4)	9.1	(8.7, 9.5)	8.5	(7.9, 9.1)	8.6	(7.9, 9.3)	8.6	(7.7, 9.4)	9.4	(8.3, 10.6)	9.9	(8.8, 10.9)
Sex																						
Female	8.8	(8.2, 9.4)	9.2	(8.5, 9.9)	8.8	(8.3, 9.4)	8.6	(7.9, 9.3)	7.7	(7, 8.5)	8	(7.4, 8.6)	7.3	(6.7, 7.8)	7.7	(6.6, 8.7)	7.2	(6, 8.4)	8.3	(7.1, 9.5)	8.3	(7.4, 9.2)
Male	10.5	(9.5, 11.5)	10.3	(9.5, 11)	10.5	(9.9, 11.1)	10.6	(9.6, 11.5)	9.9	(9.3, 10.4)	10.1	(9.3, 10.8)	9.5	(8.7, 10.4)	9.2	(8.4, 10.1)	9.8	(8.9, 10.7)	10.4	(8.7, 12)	11.1	(9.5, 12.8)
Grade																						
9th Grade	10.1	(8.7, 11.5)	9.4	(7.9, 11)	9	(8.2, 9.9)	9.7	(7.8, 11.5)	8.5	(7.4, 9.6)	9.7	(8, 11.4)	9.2	(7.8, 10.6)	9.6	(7.8, 11.4)	9.9	(8.4, 11.4)	9.5	(6.5, 12.6)	7.8	(5.6, 9.9)
10th Grade	9.1	(8.1, 10.1)	9.3	(8, 10.6)	9.6	(8.7, 10.5)	8.5	(7.5, 9.5)	7.8	(6.8, 8.8)	8.8	(7.8, 9.8)	8.4	(7.2, 9.6)	8.8	(7.3, 10.4)	9.1	(7.2, 10.9)	10.7	(9.1, 12.3)	11	(8.5, 13.5)
11th Grade	9.4	(8.8, 10.1)	9	(8, 10.1)	9.5	(8.8, 10.3)	9.1	(8, 10.1)	9	(8, 10)	8.2	(7.4, 9)	8.7	(7.8, 9.6)	8.4	(7.3, 9.5)	7.6	(6.4, 8.9)	7.8	(6.2, 9.4)	7.9	(6.1, 9.7)
12th Grade	10.3	(9.2, 11.4)	10.8	(9.8, 11.8)	10.2	(9.6, 10.7)	10.8	(10.1, 11.5)	9.4	(8.5, 10.3)	9.6	(8.9, 10.4)	8.2	(7.4, 9)	8.1	(7, 9.2)	8.5	(6.8, 10.3)	8.6	(7.2, 10.1)	10.6	(9.3, 11.9)
Race/Ethnicity																						
NH White	9.9	(9.4, 10.5)	10.1	(9.2, 10.9)	9.8	(9.4, 10.3)	9.1	(8.5, 9.6)	9.1	(8.6, 9.6)	9	(8.4, 9.5)	8.2	(7.6, 8.7)	8.4	(7.7, 9.2)	8.7	(7.9, 9.6)	8.1	(7.2, 9.1)	8.8	(7.4, 10.3)
NH Black	7.4	(6, 8.7)	6.3	(4.4, 8.2)	8.7	(7.1, 10.4)	8.8	(7.2, 10.4)	6.1	(5.2, 7.1)	7.7	(6.2, 9.2)	10	(6.3, 13.6)	8.4	(6.2, 10.7)	6.2	(4.4, 8)	9.5	(6.5, 12.5)	9.7	(6.9, 12.6)
Hispanic	7.8	(6.5, 9.2)	8.9	(7.6, 10.2)	8.9	(7.5, 10.3)	10.1	(8.6, 11.5)	8.9	(6.4, 11.3)	10.2	(8.2, 12.2)	9.6	(7.8, 11.4)	9	(7.2, 10.7)	9.7	(7.8, 11.5)	12	(9, 15.1)	11.9	(9.3, 14.5)
NH Asian	9.5	(6.1, 12.8)	7.1	(5.6, 8.5)	9.8	(5.7, 13.8)	14.5	(10.5, 18.5)	6.5	(2.1, 10.8)	10.1	(5.9, 14.4)	14.1	(10.7, 17.5)	11.5	(5.5, 17.4)	16.8	(12.3, 21.3)	13.3	(9.3, 17.4)	8	(8, 8)
NH Other	12.4	(9.9, 14.8)	10.2	(7.9, 12.4)	8.6	(7.1, 10.1)	15.4	(13.1, 17.7)	9	(7.2, 10.8)	8.1	(5.9, 10.3)	8.6	(6.5, 10.7)	9.3	(7.1, 11.5)	5.1	(2, 8.2)	13.4	(10, 16.8)	11.7	(8.9, 14.4)
